# Study of RP HPLC Retention Behaviours in Analysis of Carotenoids

**DOI:** 10.1007/s10337-014-2657-1

**Published:** 2014-03-20

**Authors:** M. Ligor, J. Kováčová, R. M. Gadzała-Kopciuch, S. Studzińska, Sz. Bocian, J. Lehotay, B. Buszewski

**Affiliations:** 1 Department of Environmental Chemistry and Bioanalytics, Faculty of Chemistry, Nicolaus Copernicus University, Gagarin Street 7, 87-100, Toruń, Poland; 2 Department of Chemistry, Faculty of Natural Sciences, University of SS. Cyril and Methodius, Trnava, Slovak Republic

**Keywords:** HPLC, Stationary phases, Retention mechanism, Carotenoids

## Abstract

For determination of selected carotenoids, various types of columns for high-performance liquid chromatography (HPLC) with different properties have been used. The characteristics of the laboratory-used packing material containing monomeric alkyl-bonded phases (C_18_, C_30_) and phenyl as well as phenyl-hexyl stationary phases were studied. The retention data of the examined compounds were used to determine the hydrophobicity and silanol activity of stationary phases applied in the study. The presence of the polar and carboxyl groups in the structure of the bonded ligand strongly influences the polarity of the stationary phase. Columns were compared according to methylene selectivity using a series of benzene homologues. The measurements were done using a methanol–water mobile phase. Knowledge of the properties of the applied stationary phase provided the possibility to predict the RP HPLC retention behaviours in analysis of carotenoids including lutein, lycopene and β-carotene. The composition of the mobile phase, the addition of triethylamine and the type of stationary phase had been taken into account in designing the method of carotenoid identification. Also a monolithic column characterised by low hydrodynamic resistance, high porosity and high permeability was applied. The presented results show that the coverage density of the bonded ligands on silica gel packings and length of the linkage strongly influence the carotenoid retention behaviours. In our study, the highest retention parameters for lutein, lycopene and β-carotene were observed for C_30_ and C_18_ stationary phase. This effect corresponds with pore size of column packing greater than 100 Å and carbon content higher than 11 %.

## Introduction

The efficiency of the separation process in reversed-phase HPLC is highly dependent on the binary hydro-organic mobile phase composition and surface properties of the packing material. The high progress in increasing the efficiency of separations of various analytes has been achieved due to introduction of a variety of column types [1, 2]. As it is commonly known HPLC packing materials with different lengths hydrophobic alkyl chains are used in analysis of environmental, pharmaceutical, biological as well as food samples. Nevertheless the separation of analytes from complex matrix poses some problems. The knowledge of the retention mechanism gives possibility to select proper chromatographic conditions and for the separation of components especially in food products.

As it was proposed by Galushko, for the characterisation of packing materials used for HPLC, the evaluation of silanol activity (SA_G_) and hydrophobicity (*H*_G_) of stationary phases is recommended [3]. To determine these parameters, the analysis of solutions containing aniline, phenol, benzene and toluene in proper HPLC conditions should be done. According to literature data [3] the silanol activity (Eq. 1) and hydrophobicity (Eq. 2) could be calculated as follows using retention factors *k* of analytes mentioned above:

1 $$ {\text{SA}}_{G} = 1 + 3\left[ {\left( {\frac{{k_{\text{anilin}} }}{{k_{\text{phenol}} }}} \right) - 1} \right] $$

2 $$ H_{G} = \left( {\frac{{k_{\text{toluene}} + k_{\text{benzene}} }}{2}} \right) $$

In reversed-phase HPLC, the binary hydro-organic mobile phase composition together with undersurface properties of the packing material influences the separation efficiency [1, 4]. The specific separation has been achieved due to introduction of a variety of column types such as cyclodextrin, aryl phases, and materials containing mixed ligands or exhibiting liquid-crystalline properties [5–8]. Researchers have used carotenoids as test substances in the process of classifying silica-based columns modified with octadecyl functional groups [9–12]. Lessellier et al. [9] determined the hydrophobicity, steric effect and surface activity of C_18_ phases. In the above study, the analysed compounds were *cis* and *trans* isomers of β-carotene and zeaxanthin and the analytical method was supercritical fluid chromatography (SFC) [10]. The discussed technique can separate analytes based on analyte-stationary phase interactions with shorter run times. Carbon dioxide, as a supercritical fluid, has many advantages, including ease of diffusion, absence of surface tension, low viscosity and the ability to dissolve analytes, which are characteristic features of liquids. SFC gives possibility for the separation of compounds containing *cis* and *trans* isomers. The use of supercritical fluid intensifies the interactions between the analysed compounds and the stationary phase in comparison with reverse-phase liquid chromatography (RPLC) [10]. SFC analyses have revealed a linear correlation between the selectivity of *cis* and *trans* isomers of β-carotene and the chain length of the stationary phase, percent coverage (% C) and coverage density.

On the other hand, a new method to improve recoveries and selectivities such as HPLC–MS–MS has been developed for the determination of β-carotene and xanthophylls in selected vegetables. In proposed HPLC–MS–MS method, an atmospheric pressure interface was used. Moreover, collision-induced dissociation (CID) in the ion source gave a possibility for the identification of carotenoids by their characteristic fragmentation pathways [13].

The compounds β-carotene and xanthophylls mentioned above belong to carotenoids, which are one of the most interesting group of biologically active compounds. Carotenoids play an important role in photosynthesis. Those photolabile compounds are susceptible to change under the influence of UV radiation. Carotenoids exert antioxidant effects and protect the organism against the harmful effects of UV radiation. They deliver significant health benefits for humans by protecting the human body from harmful external factors (solar radiation, environmental pollution). Plants (fruit and vegetables) are the main source of carotenoids in the human diet. Carotenoids, including xanthophylls, belong to a large group of natural pigments. The chemical structure of 600 compounds has been successfully described to date. Today, 200 carotenoids are chemically synthesised [14–16].

Carotenoid pigments are classified into three groups:

1. Carotenes: hydrocarbons whose molecules contain 11 conjugated double bonds in the all-*trans* configuration; empirical formula C_40_H_56_.
2. Xanthophylls: oxygen derivatives of carotenes whose molecules contain hydroxyl, epoxy or carbonyl groups.
3. Apo-carotenoids: compounds containing <40 carbon atoms per molecule, where the central fragment of the carotene molecule with four methyl groups has been preserved.

The structure of carotenoids is made up of eight isoprene units joined in such a way that isoprene residues are connected head to tail in the centre of the molecule [14]. Two methyl groups near the centre of the molecule are found at positions 1–6, whereas the remaining methyl groups are situated at positions 1–5.

The characterization and structures of selected carotenoids, which were taken under consideration in this study, are presented in Table 1.

**Table 1:** Structure of selected carotenoids

Compound	Chemical class	Structure	CAS number	Molecular weight MW (g mol^−1^)
Lutein	Xanthophylls		127-40-2	568.87
Lycopene	Carotenes		502-65-8	536.87
β-Carotene	Carotenes		7235-40-7	536.87

A characteristic series of conjugated double bonds creates a chromophore system. The presence of double bonds conditions geometric isomerism. *Trans* isomers are easily transformed into *cis* isomers under the influence of light and temperature. *Cis* isomers are generally less intensely coloured than *trans* isomers, they absorb UV radiation more readily and demonstrate lower levels of biological activity. Carotenoids’ ability to absorb UV radiation results from the number of conjugated double bonds in a molecule. The presence of carbonyl and carboxyl groups in a molecule determines the colour intensity of a given carotenoid [15]. The presence of conjugated double bonds in carotenoids molecules gives possibility to obtain numerous degradation products. High ambient temperature promotes and accelerates the carotenoids degradation process. Also, it was noted that a small amount of products contain oxygen in molecule structure. This is probably caused by connection of oxygen to double bonds.

The objective of this study was the description of the RP HPLC retention mechanism of carotenoids on the ground of applied stationary phase properties. Obtained data gave a possibility to select stationary phases and suitable chromatographic conditions of the carotenoids separation process. The results of the analysis provide valuable information about compound retention and the separation effect. The composition of the mobile phase, the addition of triethylamine (TEA) and the type of stationary phase had been taken into account in designing the method of carotenoid identification. The retention data of the examined compounds were used to determine the hydrophobicity of stationary phases applied in the study.

## Experimental

### HPLC Instrumentation and Conditions

The analysis was performed with an Agilent separation module 1100 series (Agilent Technologies, Palo Alto, CA, USA) equipped with UV–VIS detector, controlled by a ChemStation Software (Agilent Technologies). To determine silanol activity and the hydrophobicity, the analysis of aniline, phenol, benzene and toluene was done using a mobile phase containing 60 % methanol in water. The concentration of each compound was 10 μg mL^−1^, respectively. The mobile phase was the composition of methanol:water (60:40 v/v) in an isocratic run. Flow rate was set at 0.5 mL min^−1^ and injection volume was 10 μL. A UV–VIS was used, and detection being at the wavelengths of absorption *λ* = 254 nm.

In an analytical procedure proposed for the separation of analytes from the mixture containing lutein, lycopene and β-carotene, the chemical properties of compounds and introduced HPLC packing materials were taken into consideration. Therefore, the mobile phase was composed by acetonitrile:methanol:ethyl acetate (v/v) in a gradient, from 95:5:0 to 60:20:20 in 20 min, the latter proportion being maintained until the end of the run. Flow rate was set at 0.5 mL min^−1^ and injection volume was 10 μL. Acetonitrile contained 0.05 % of triethylamine to improve carotenoid recovery from the chromatographic column. A UV–VIS was used, and detection being at the wavelengths of maximum absorption *λ* = 450 nm.

### Chemicals

To determine the silanol activity and hydrophobicity of tested stationary phases, standard compounds such as aniline, phenol, benzene and toluene (≥99.5 % purity) (Sigma-Aldrich, St. Louis, MO, USA) were used for the preparation of methanolic solution. Solvents such as acetonitrile, methanol and ethyl acetate (≥99.9 % purity) were purchased from Sigma-Aldrich (St. Louis, MO, USA). For the analytical procedure standard mixtures of carotenoids including lutein (≥95.0 % purity), obtained from Carl Roth GmbH, Karlsruhe, Germany, lycopene and β-carotene (≥97.0 % purity), obtained from Sigma-Aldrich (St. Louis, MO, USA), in methanol were prepared. Carotenoids such as lutein, lycopene and β-carotene at a concentration 100 μg mL^−1^ of each compound were analysed by means of HPLC.

### Physico-Chemical Values and Selectivity Data of Columns

The list of used columns is available in Table 2. The parameters of chromatographic columns used in this study such as dimensions, particle size, medium, end-capping (EC) information as well as particular specification are presented. Most of phases studied were monomeric alkyl-bonded phases, in particular 11 silica gel columns with an octadecyl-bonded phase (C_18_), 1 column with C_30_, also 1 phenyl and phenyl-hexyl stationary phase, as well as 1 monolithic column.

**Table 2:** The list of used columns, physico-chemical values and selectivity data

Column	Manufacturer	Dimensions (mm)	Particle size (*d*_p_) (μm)	Medium	Stationary phase	End-capping (EC)	Specification		
							Pore size (Å)	Specific surface area (*S*_BET_) (m^2^ g^−1^)	Carbon content (%)
Discovery C_18_	Supelco	150 × 4.6	5.0	Silica gel	Octadecyl	+	180	200	12
Halo C_18_	Advanced Materials Technology	100 × 3	2.7	Silica gel	Octadecyl	+	90	150	−
XBridge C_18_	Waters	150 × 4.6	3.5	Silica gel	Octadecyl	+	130	185	18
GraceSmart RP 18	Alltech	100 × 2.1	3.0	Silica gel	Octadecyl	+	120	220	−
Betasil C_18_	Thermo	50 × 4.6	5.0	Silica gel	Octadecyl	+	100	−	20
Kinetex C_18_ 100A	Phenomenex	100 × 3	2.6	Silica gel	Octadecyl	+	100	200	12
Ib-Sil C_18_	Phenomenex	50 × 3.2	5.0	Silica gel	Octadecyl	+	125	165	−
Onyx Monolithic C_18_	Phenomenex	100 × 4.6		Monolith	Octadecyl	−	130	300	18
Platinum C_18_ 100A	Alltech	150 × 4.6	5.0	Silica gel	Octadecyl	+	100	200	6
Spherisorb	Waters	50 × 4.6	3.0	Silica gel	Phenyl	−	80	220	2.5
Spherisorb ODS2	Waters	125 × 4.6	5.0	Silica gel	Octadecyl	+	80	220	11.5
X SELECT	Waters	150 × 3.0	3.5	Silica gel	Phenyl-hexyl	+	130	185	14
Spherisorb ODS-2	Carl Stuart Ltd	250 × 4.6	3.0	Silica gel	Octadecyl	+	80	220	11.5
ProntoSIL	BISCHOFF	250 × 4.6	3.0	Silica gel	C_30_	−	120	300	25
Hichrom	Labquip	250 × 4.4	10.0	Silica gel	Octadecyl	+	100	200	11

### Statistical Data Analysis

Principal component analysis (PCA) and cluster analysis (CA) were performed on a personal computer employing a Statistica Package v. 8.0 (StatSoft, Tulsa, OK, USA).

## Results and Discussion

The applied columns contained modified silica gel with particle size (*d*_p_) from 2.6 to 10.0 μm. Specific surface area (*S*_BET_) of packing ranged from 150 to 300 m^2^ g^−1^. The percentage content of carbon varied subject to the type of stationary phase, and it reached 25 % for column C_30_, from 2.5 to 20 % for column C_18_, 18 % for a monolithic column, 14 % for a phenyl-hexyl column and 2.5 % for a phenyl column. Polymer-based monolithic columns pose an alternative for silica gel which is used as a stationary phase in column chromatography. Monolithic columns are characterised by low hydrodynamic resistance, high porosity and, consequently, high permeability. The monolith technology allows the use of high flow rates for faster analyte separations [17, 18].

The knowledge about hydrophobic or lipophilic character of biologically active compounds is important for the development of their methods of determination. Molecules of hydrophobic compounds usually do not have a dipole moment. These compounds can be considered as non-polar. Some compounds can be both hydrophilic and hydrophobic, because their molecules belong to both polar and non-polar groups. Also, stationary phases used in chromatography can be hydrophobic [19, 20], while the lipophilicity of compounds determines the biological action of them. With regard to living organism, this parameter affects the solubility of the compound, and consequently its bioavailability and distribution in the body. Various compounds penetrating or permeating into organism cells surmount phospholipid layers (called barriers). Lipophilic biological membrane can be defeated only by molecules of selected compounds, characterised by high chemical affinity, which is connected with their effective distribution within the organism. The mechanism of permeation of biologically active compounds through biological membranes can be similar to that during chromatographic separation. Therefore, an evaluation of hydrophobic properties of stationary phases was the important part of the study.

Characterisation of stationary phases was done by the evaluation of their silanol activity and hydrophobicity. To determine SA_G_ and *H*_G_ parameters, the analyses of mixture containing aniline, phenol, benzene and toluene are recommended. The retention data of four standard compounds on the tested stationary phases were compared using mobile phase containing 60 % methanol in water. The results are presented in Table 3.

**Table 3:** The stationary phase silanol activity and hydrophobicity evaluated for standards by means of Galushko’s test

No.	Column	Retention factor *k* ^a^				Silanol activity	Hydrophobicity
		Aniline	Phenol	Benzene	Toluene		
1.	Discovery C_18_	0.262 ± 0.013	0.387 ± 0.019	1.661 ± 0.083	3.263 ± 0.163	0.029	2.46
2.	Halo C_18_	0.223 ± 0.011	0.326 ± 0.016	1.732 ± 0.087	3.618 ± 0.181	0.051	2.67
3.	XBridge C_18_	0.346 ± 0.017	0.508 ± 0.025	2.227 ± 0.111	4.328 ± 0.216	0.045	3.28
4.	GraceSmart RP 18	0.139 ± 0.007	0.158 ± 0.008	0.595 ± 0.030	1.349 ± 0.067	0.628	0.97
5.	Betasil C_18_	0.434 ± 0.022	0.617 ± 0.031	2,934 ± 0.147	5.902 ± 0.295	0.112	4.42
6.	Kinetex C_18_ 100A	0.192 ± 0.010	0.274 ± 0.014	1.195 ± 0.060	2.321 ± 0.116	0.103	1.76
7.	Ib-Sil C_18_	0.187 ± 0.009	0.223 ± 0.011	1.076 ± 0.054	2.189 ± 0.109	0.522	1.63
8.	Onyx Monolithic C_18_	0.193 ± 0.010	0.256 ± 0.013	1.203 ± 0.060	2.357 ± 0.118	0.267	1.78
9.	Platinum C_18_ 100A	0.246 ± 0.012	0.262 ± 0.013	1.094 ± 0.055	1.993 ± 0.100	0.810	1.54
10.	Spherisorb	0.173 ± 0.009	0.144 ± 0.007	0.345 ± 0.017	0.466 ± 0.023	1,620	0.41
11.	Spherisorb ODS2	0.465 ± 0.023	0.555 ± 0.028	2.391 ± 0.120	4.566 ± 0.228	0.517	3.48
12.	X SELECT	0.315 ± 0.016	0.397 ± 0.020	1.049 ± 0.052	1.791 ± 0.090	0.378	1.42
13.	Spherisorb ODS-2	0.340 ± 0.017	0.374 ± 0.019	1.675 ± 0.084	3.222 ± 0.161	0.729	2.45
14.	ProntoSIL	0.394 ± 0.020	0.422 ± 0.021	1.659 ± 0.083	3.201 ± 0.160	0.803	2.43
15.	Hichrom	0.383 ± 0.019	0.385 ± 0.019	1.206 ± 0.060	2.033 ± 0.102	0.989	1.62

^a^ *k* values are presented as mean value ± SD (*n* = 3)

Among octadecyl phases the highest hydrophobicity (ranging from 2.46 to 4.42) was evaluated for columns like Betasil C_18_, Spherisorb ODS2, XBridge C_18_ and Discovery C_18_. For ProntoSIL, where the stationary phase in the column are long alkyl chains (C_30_) this parameter amounted only to 2.43. In the case of columns: Onyx Monolithic C_18_, XSelect and Spherisorb, we obtained hydrophobicity 1.78; 1.42 and 0.41, respectively. On the contrary, the highest silanol activity (1.620) for the column Spherisorb with phenyl stationary phase was characterised. Spherisorb column was filled with adsorbent that was not subjected to a secondary silylation process (called end-capping), which could result in higher values of SA_G_ than other columns. In addition, Spherisorb column was characterised by the lowest percentage of carbon content (2.5 %), which results in the possibility of interaction between analytes and free silanol groups on the surface of silica gel. Lower SA_G_ values were found for columns Hichrom (0.989), Platinum C_18_ 100A (0.810) and ProntoSIL (0.803). In general, for columns used in this study, the hydrophobicity of stationary phase decreased with an increase of silanol activity.

The development of advanced HPLC technique gives new possibilities in the analysis of carotenoids [13]. Therefore, the knowledge of retention behaviour of these compounds is an important problem. Mentioned compounds such as lutein, lycopene and β-carotene belong to the hydrophobic compounds, according to Nernst’s law, in the separation process of the carotenoid in the RP HPLC, followed by the partition of the analyte between mobile phase and stationary phase in the column. In case of columns filled C_18_ stationary phase Van der Waals interactions could be expected for carotenoids. A predominance of chain ⇔ chain interactions could also be encountered between octadecyl chains of the stationary phase and carbon chains of various carotenoids.

**Fig. 1:**
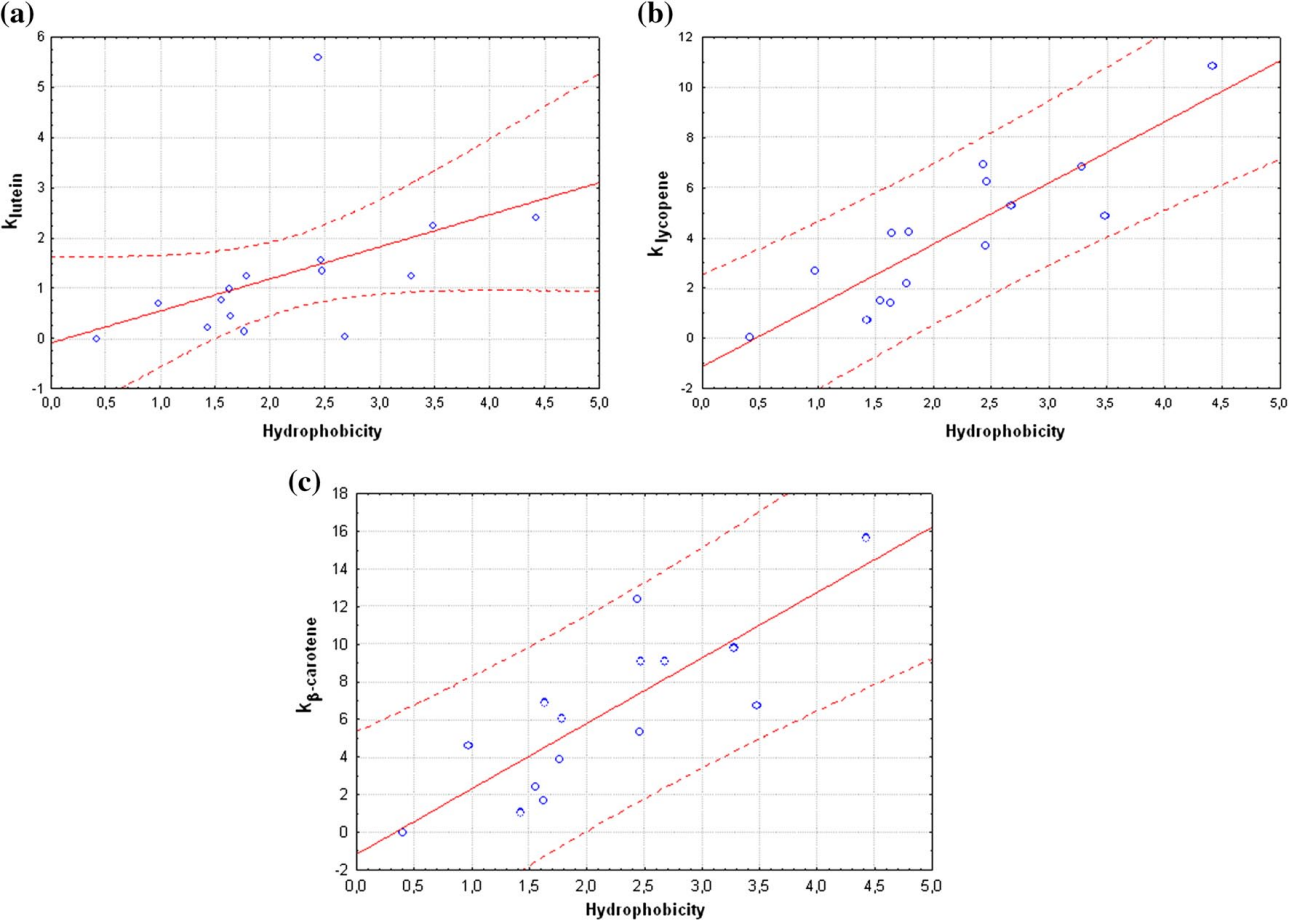
Charts of retention factors of lutein (**a**), lycopene (**b**) i β-carotene (**c**) vs. hydrophobicity of stationary phase

Depending on the retention factors obtained for selected carotenoids and hydrophobicity values for studied stationary phases, mathematical relationships were computed. Appropriate charts prepared using Statistica Package are presented in Fig. 1. The dispersion of retention factors of lutein, lycopene and β-carotene in relation of the hydrophobicity of stationary phases was taken into consideration.

Among the selected carotenoids, β-carotene and lycopene are more hydrophobic than lutein. There are two hydroxyl groups in molecule structure of lutein. The presence of two polar hydroxyl groups on opposite ends of the molecules influences a significantly lower hydrophobic of this compounds. In the case of β-carotene and lycopene high correlation between retention factors of these compounds and hydrophobicity of stationary phases have been observed. This indicates that the chromatographic retention mechanism of these compounds may be based on hydrophobic interactions. Measuring points on the charts, obtained for lycopene and β-carotene (b and c in Fig. 1) are located in a 95 % confidence interval. In the case of lutein a different situation was observed. The presence of the hydroxyl groups in the molecule caused changes in the mechanism of retention compared to β-carotene and lycopene. For three columns (ProntoSIL, Kinetex C_18_ 100A, Halo C_18_), obtained results do not fall within the confidence interval set at the level of 95 %. It confirms the significant impact of the hydroxyl groups on the retention mechanism of lutein in column filled with hydrophobic stationary phase. The largest deviation has been observed in the case of ProntoSIL, which is usually applied to analysis of carotenoids, due to relatively high hydrophobicity of C_30_ stationary phase. Long hydrophobic chains of C_30_ stationary phase effectively cover reactive free silanol groups on the surface of silica. Presented data confirm the fact that the retention mechanism of lycopene and β-carotene is based on hydrophobic effect, while in the case of lutein hydrophobic interactions are weakened due to the presence of the hydroxyl groups. The presence of the hydroxyl groups in the lutein structure significantly reduces retention factor of this compound. In case of hydrophobic stationary phase, during chromatographic process polar components in mobile phase are displaced from among hydrophobic organic ligands of stationary phase.

**Fig. 2:**
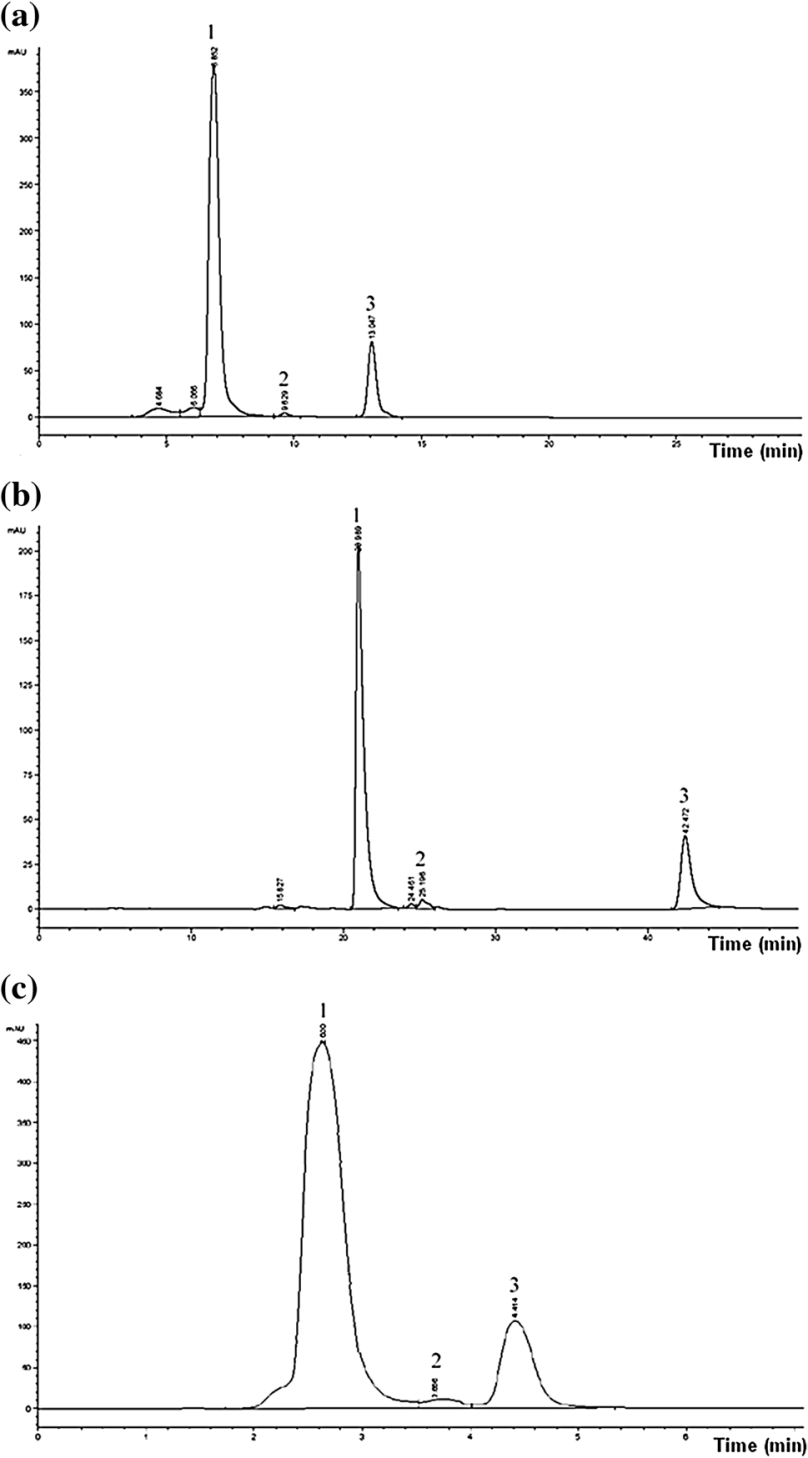
Chromatograms of mixture containing lutein (*1*), lycopene (*2*) and β-carotene (*3*) obtained by HPLC–UV–VIS using the following columns: **a** C_18_, **b** C_30_, **c** phenyl-hexyl

The examined compounds’ retention times were determined by the type of stationary phase, its hydrophobicity, column length, specific surface area and pore size. Exemplary chromatograms for C_18_, C_30_ and phenyl-hexyl columns are presented in Fig. 2.

Retention parameters for various analytes were compared based on the values of retention factors.

**Fig. 3:**
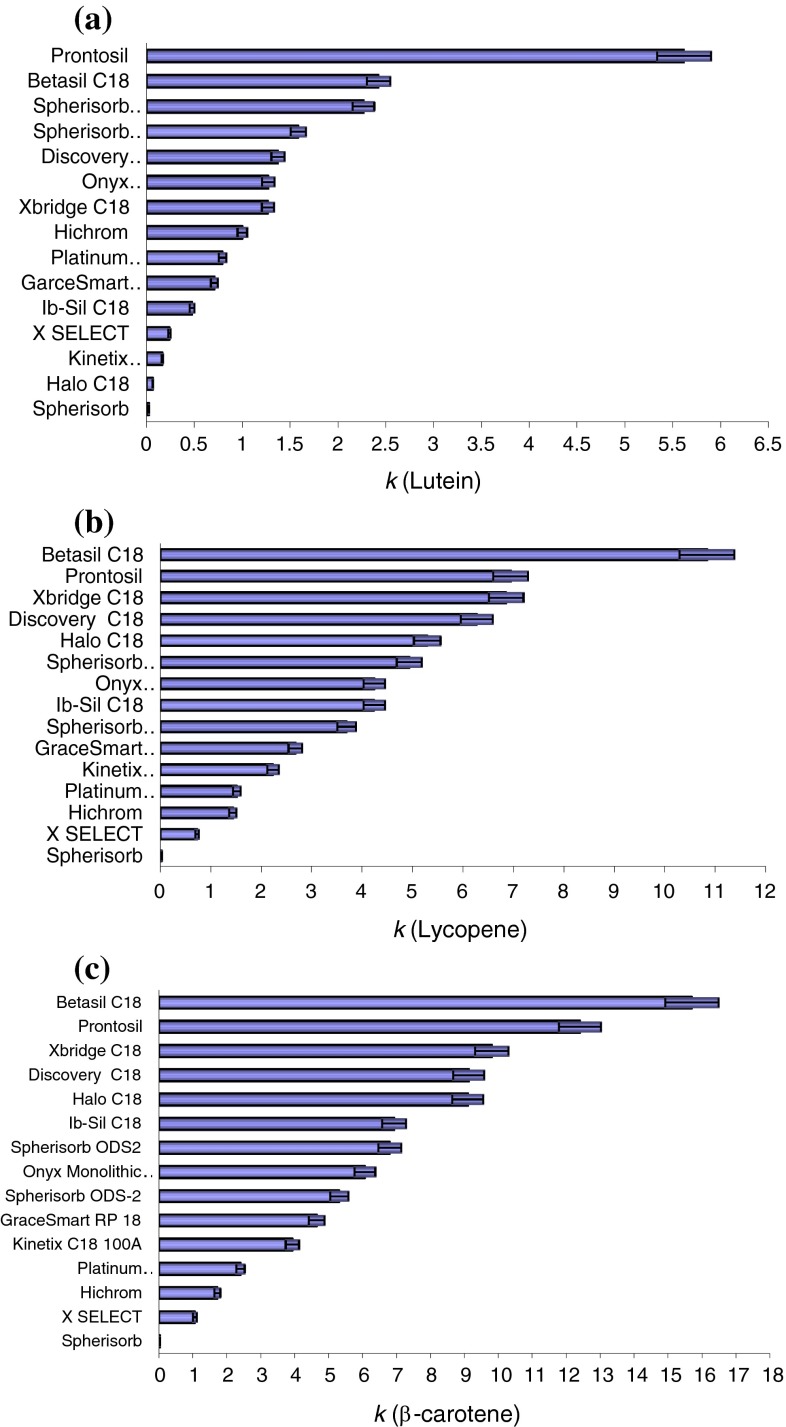
Correlations between changes in retention factor (*k*) and the type of stationary phase, determined for **a** lutein, **b** lycopene and **c** β-carotene

The results of calculated retention factors and columns ranked according to retention for lutein, lycopene and β-carotene are presented in the form of histograms in Fig. 3.

Carotenoids can also be used as a probe to measure a hydrophobicity of stationary phases. Notice that the ranking of applied columns according to retention for lutein is slightly different from the ranking according to retention for lykopene and β-carotene (Fig. 3). In our study, the highest retention parameters for lutein, lycopene and β-carotene were reported for columns with pore size >100 Å and carbon content higher than 11 %. The above results were reported in the following columns: ProntoSIL (C_30_ stationary phase) where retention factors for lutein, lycopene and β-carotene were noted at 5.614, 6.941 and 12.395, respectively; Betasil C_18_ at 2.423, 10,835 and 15.691, respectively; XBridge C_18_ at 1.267, 6.854 and 9.794, respectively. Spherisorb (phenyl stationary phase), X SELECT (phenyl-hexyl stationary phase) and Hichrom (ODS—octadecylsilane) columns failed to separate the analysed compounds or the analytes were not completely separated.

Eluent used as a mobile phase composed of a solvent mixture contained in the initial gradient—methanol (5 %) and acetonitrile (95 %). Under the above conditions, stationary phases containing octadecyl chains may undergo phase collapse [21]. The components of the analysed mixture are not separated into the stationary phase, and they are quickly eluted from the column. The above process produces low retention factors for the investigated analytes. Higher retention factors were reported for longer columns and end-capped columns. The studied compounds were characterised by stronger hydrophobic interactions with the stationary phase. Symmetrical peaks of the analysed substances were obtained in end-capped columns. The observed values of the peak asymmetry factor did not exceed *f*_AS_ = 1.0 ± 0.1. One of the analysed phases was capable of bridging residual silanol groups (XBridge C_18_ column). This chemical structure prevents the hydrolysis of bonds between silica and the alkyl chain, thus improving the chemical stability and mechanical strength of packing. Nonetheless, when applied in XBridge C_18_ columns, the above modification increased the asymmetry factor (above 1.0 for lycopene and 0.9 for β-carotene).

The addition of triethylamine to the mobile phase had a positive impact on peak symmetry. In most cases the asymmetry factors (*f*_AS_) of determined compounds were slightly reduced (e.g. for β-carotene from 1.1 to 0.9). Also the addition of TEA reduced the peak tailing effect. The process of TEA addition is also referred as dynamic modification of the stationary phase. Free silanol groups on the surface of silica are blocked when they interact with alkaline molecules of triethylamine. The above also prevents the analyte from interacting with the medium, i.e., free silanol groups on the surface of silica. The use of TEA significantly influences substance retention. The noted effects include longer analyte retention times and hydrolysis of the silica bed [21, 22]. The addition of triethylamine to the mobile phase can also have negative consequences, such as changes in the pH of the mobile phase, therefore, it is recommended that TEA is used in amounts corresponding to the concentration of 0.05 %.

**Fig. 4:**
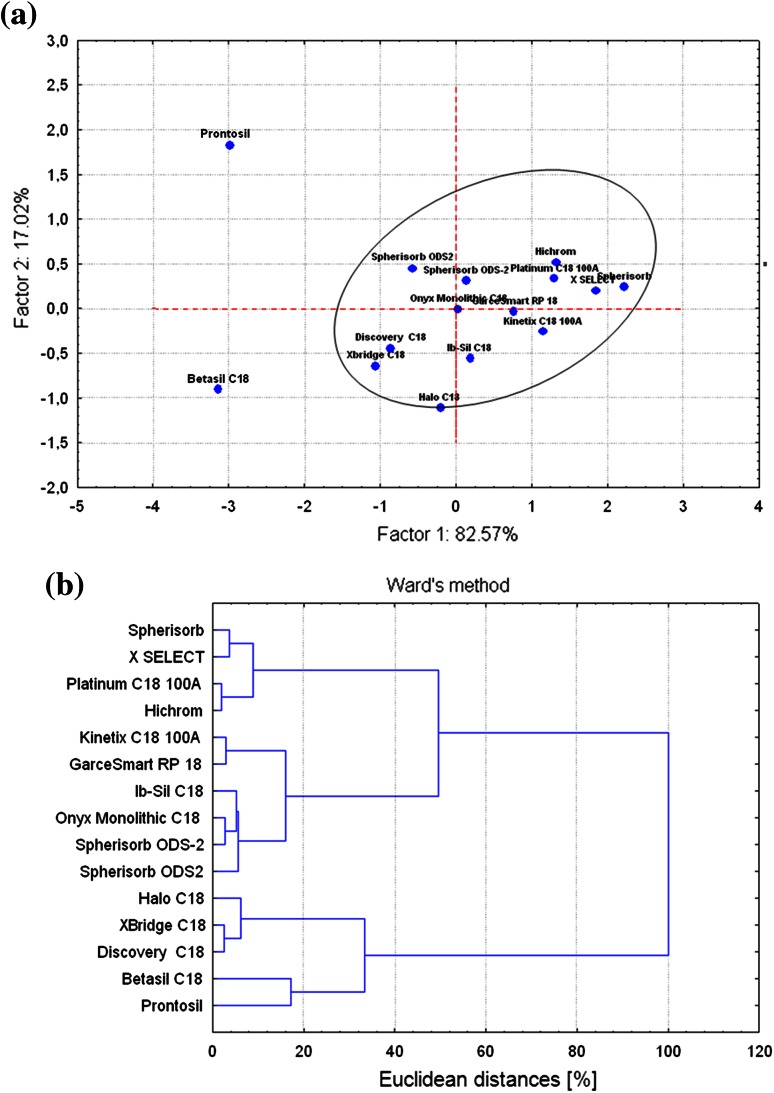
Two-dimensional scatter plots of the loadings of the first two factors PCs (PC1 and PC2) for lutein, lycopene and β-carotene retention factors by use of fifteen HPLC columns; **a** PCA and **b** CA diagram (Ward’s method)

Similarities and differences between investigated types of HPLC columns were evaluated by chemometric methods using retention factors of analysed carotenoids. The differences between columns are explained by the PCA and CA analyses (Fig. 4). Previously obtained retention factors of lutein, lycopene and β-carotene gave possibilities to the analysis of the principal components. The PCA and CA were obtained by means of Statistica Package v. 8.0. In Fig. 4a, projection of fifteen HPLC columns onto the plane of the two first principal components, PC1 and PC2, is presented. Obtained results, remaining statistically valuable for the consecutive PCA analysis, indicate also the possible differences between the columns used on the physico-chemical level.

PCA allows for the comparison of retention data obtained for stationary phases used in this study. We observed that two columns ProntoSIL and Betasil C_18_ do not meet the same criteria as others. It should be mentioned that the retention factors, obtained for lutein, lycopene and β-carotene, were highest when ProntoSIL (C_30_ stationary phase) and Betasil C_18_ were applied. These results can be connected to a high content of carbon and specific surface area of mentioned stationary phases (Table 2).

Chromatographic pattern described by retention data was processed using classification method CA. Cluster analysis was used to distinguish characteristic of stationary phases. A dendrogram was charted to represent relations between different columns (Fig. 4b). Three groups of columns can be discriminated. The first one consists of Spherisorb, X SELECT, Platinum C_18_ 100A, and Hichrom. The second one contains Kinetex C_18_ 100A, GraceSmart RP 18, Ib-Sil C_18_, Onyx Monolithic C_18_, as well as two columns Spherisorb ODS-2 and Spherisorb ODS2. An interesting aspect was observed for this group. It is characterised by small differences within this group of column. The third group consists column such as: Halo C_18_, XBridge C_18_, Discovery C_18_, Betasil C_18_, ProntoSIL. Dissimilarities between columns in this group are rather high, therefore subdivision of it is possible on 20 % of maximal relative Euclidean distance. In order to make a more complete characterization of stationary phases, it was necessary to carry out further research with the use of other substances.

Moreover selectivity factors for mentioned columns were computed. Obtained results are presented in Table 4. Relative retention of analyte, also known as the selectivity factor or separation factor (*α*), is described by the separation of two species (A and B) on the column. When calculating the selectivity factor, species A elutes faster than species B and this parameter is always greater than one.

**Table 4:** Selectivity factors determined for HPLC columns with various stationary phases

Column	*α* = *k*_lycopene_/*k*_lutein_	*α* = *k*_β-carotene_/*k*_lycopene_
Discovery C18	4.568	1.455
Halo C18	82.026	1.720
XBridge C18	5.410	1.429
GraceSmart RP 18	3.782	1.736
Betasil C18	4.472	1.448
Kinetix C18 100A	13.579	1.760
Ib-Sil C18	8.925	1.633
Onyx monolithic C18	3.334	1.430
Platinum C18 100A	1.905	1.586
Spherisorb	1.000	1.000
Spherisorb ODS2	2.176	1.378
X SELECT	3.048	1.454
Spherisorb ODS-2	2.327	1.439
Prontosil	1.236	1.786
Hichrom	1.434	1.197

**Fig. 5:**
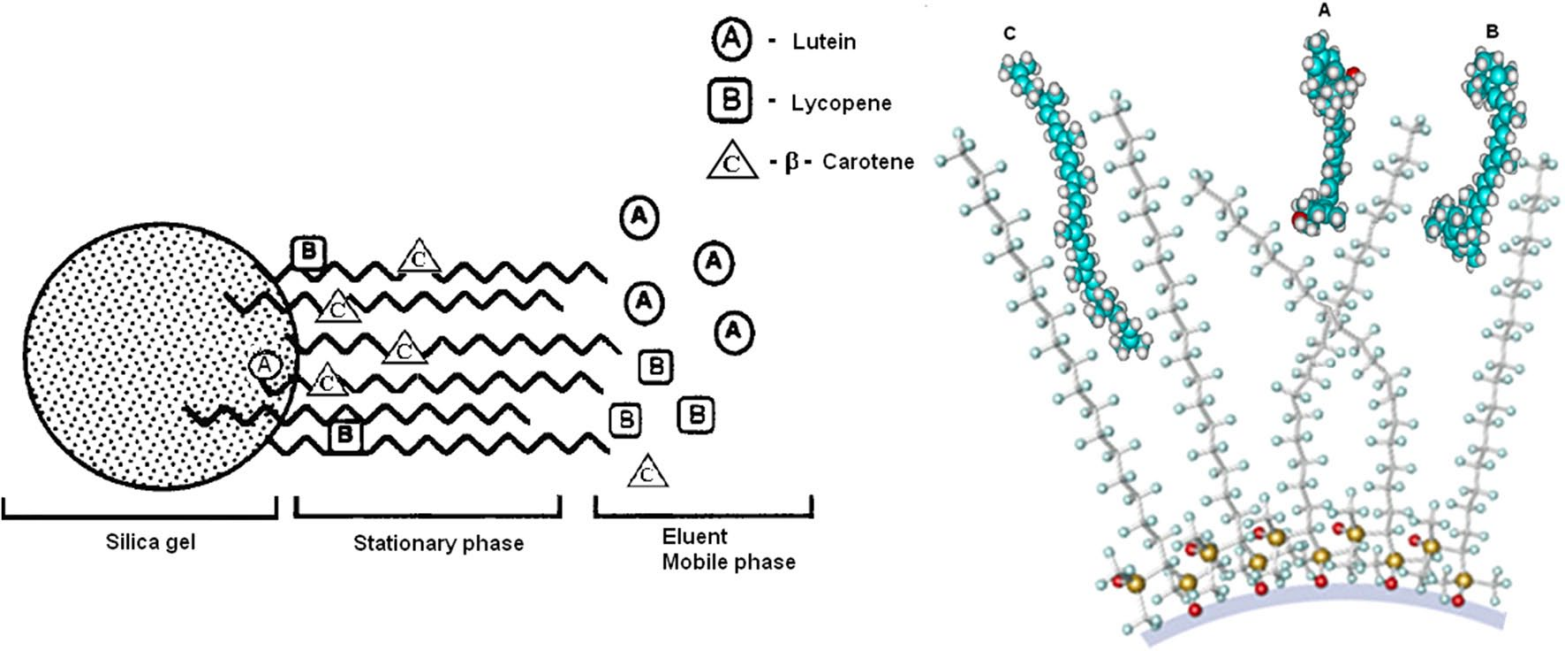
Scheme of mechanism of carotenoid separation processes by use of RP HPLC; two cases: C_18_ stationary phase without EC (**1**) and with an inactivation of free silanols (**2**); lutein (*A*), lycopene (*B*) and β-carotene (*C*)

The structure of carotenoids analysed in this experiment contains aromatic rings and non-polar carbon chains of isoprene units that form series of conjugated double bonds. The above significantly influences the type of interactions between carotenoids and the stationary phase. A lutein particle contains polar hydroxyl groups which could be responsible for hydrogen and donor–acceptor interactions with the stationary phase. Aromatic rings condition the formation of *π*–*π* interactions, whereas hydrophobic chains contribute to van der Waals interactions. In Fig. 5, the schematic separation of carotenoids in the RP HPLC is presented. Two cases are shown: column bonded with C_18_ stationary phase and inactivated free silanols and C_18_ column without EC. Molecules of organic solvents occurring in eluent are not included.

More hydrophobic analytes such as lycopene and β-carotene are more attracted to the hydrophobic bonded phase. Moreover, these compounds spend more time associated with the bonded phase than lutein and are eluted last. Under the same analysis conditions the retention of lycopene will be longer. However, the spatial structure of this compound causes the occurrence of interactions with bonded ligands of the stationary phase will also be limited. In addition, the presence of solvent effects, causing the shielding of the stationary phases by the molecules of the solvent, limits the possibility of hydrophobic interactions between lycopene and the stationary phase. In the case of β-carotene, both the structure of this compound and arrangement of ligands of column stationary phase, even when there is a high coverage density of bonded ligands, allow the free penetration of β-carotene into the stationary phase. It resulted the highest retention time of β-carotene. The occurrence of solvatation effects and the presence of free silanol groups have no effect on the β-carotene retention. In general, the retention mechanism of compounds including carotenoids may be divided for various types of interaction which results from their molecular properties, length and density coverage of bonded ligands of stationary phase as well as mechanism of the interaction with stationary and mobile phase [23, 24]. Hydrophobic part of the molecule interacts with organic ligands, whereas polar atoms (oxygen) may interact with silanol via hydrogen bonding. For this reason, the stationary phase with lower surface coverage may be more effective for chromatographic determination of these compounds in RP HPLC.

## Conclusions

The majority of columns analysed in this study were packed with octadecyl chemically bonded to silica gel. For columns with deactivated stationary phase (end-capping), the presence of polar interactions between a silica packing and analytes will be largely restricted. The above indicates that retention efficiency will be determined by hydrophobic interactions. The addition of TEA to the mobile phase had a positive impact on peak symmetry and reduced the peak tailing effect. Stationary phases with high specific surface area (200–200 m^2^ g^−1^) and high carbon content (≥11 %) will generally retain hydrophobic compounds longer than phases with low surface area and low carbon content. During analysis of carotenoids it was observed that a wider pore of stationary phase (≥100 Å) usually provides better performance, than a phase with small pores. High purity silicas provide better peak shape for compounds than more acidic silicas. However, stationary specifications will not give users enough information to accurately predict retention or selectivity, in particular when polar compounds are separating.

The presented results indicate that a efficient separation of lutein, lycopene and β-carotene in relatively short analysis time is obtained by use of HPLC column with C_18_ stationary phases. The highest retention parameters for carotenoids were observed for C_30_ stationary phase. It corresponds with pore size of column packing (120 Å) and carbon content (25 %). The high correlation between retention factors of β-carotene and lycopene and hydrophobicity of stationary phases has been observed. It is highly probable that the chromatographic retention mechanism of these compounds based on hydrophobic interactions. In the case of lutein hydrophobic interactions are weakened due to the presence of the hydroxyl groups. It resulted the significant reduction of lutein retention factor during HPLC analysis.
